# pH-Activated Dissolvable Polymeric Coatings to Reduce Biofouling on Electrochemical Sensors

**DOI:** 10.3390/jfb14060329

**Published:** 2023-06-20

**Authors:** Ahmet Uçar, Eva González-Fernández, Matteo Staderini, Alan F. Murray, Andrew R. Mount, Mark Bradley

**Affiliations:** 1 School of Engineering, Institute for Bioengineering, The University of Edinburgh, The King’s Buildings, Mayfield Road, Edinburgh EH9 3JL, UK; alan.murray@ed.ac.uk; 2 Department of Energy Systems Engineering, Faculty of Engineering and Natural Sciences, Ankara Yıldırım Beyazıt University, 06010 Ankara, Turkey; 3 School of Chemistry, University of Edinburgh, Joseph Black Building, West Mains Road, Edinburgh EH9 3FJ, UK; gfernandezeva@hotmail.com (E.G.-F.); mat.staderini@gmail.com (M.S.); a.mount@ed.ac.uk (A.R.M.)

**Keywords:** dissolvable packaging, pH-activation, biofouling, electrochemical sensing, implantable devices

## Abstract

Implantable electrochemical sensors that enable the real-time detection of significant biomarkers offer huge potential for the enhancement and personalisation of therapies; however, biofouling is a key challenge encountered by any implantable system. This is particularly an issue immediately after implantation, when the foreign body response and associated biofouling processes are at their most active in passivating a foreign object. Here, we present the development of a sensor protection and activation strategy against biofouling, based on coatings consisting of a pH-triggered, dissolvable polymer, that covered a functionalised electrode surface. We demonstrate that reproducible delayed sensor activation can be achieved, and that the length of this delay can be controlled by the optimisation of coating thickness, homogeneity and density through tuning of the coating method and temperature. Comparative evaluation of the polymer-coated and uncoated probe-modified electrodes in biological media revealed significant improvements in their anti-biofouling characteristics, demonstrating that this offers a promising approach to the design of enhanced sensing devices.

## 1. Introduction

There has been widespread interest in the development of electrochemical sensors and biosensors for the selective detection of different analytes, both in vitro and in vivo. This includes their integration into wearable [1,2] or implantable [3,4] platforms, due to their high sensitivities, low detection limits, modifiable electrode architectures, and ready integration, with control and analysis instrumentation along with minimal sample requirements. However, when an implanted device needs to be used for long periods of time, biofouling often causes the sensor transducer (functionalised electrode) surface to become passivated by fibrous capsules and non-specific proteins [5]. This foreign-body response remains a major issue to be addressed for implantable electrochemical sensing devices as it significantly limits their lifetime and compromises their response [6]. Indeed, if implanted biomaterials/devices are not suitably designed or protected, potential risks related to the foreign body response include capsule formation, prolonged inflammation, haemorrhage, tissue oedema, fibrosis, synovitis, thrombosis, restenosis and organ failure [7]. Accordingly, there is growing interest in avoiding or blocking biofouling through minimising the size of the sensor, reducing material-tissue interactions and/or protecting these implantable sensing systems from tissue exposure. This includes a variety of approaches based on polymers, including hydrogels, SAMs, MEMS and even fluidic systems [8,9,10].

Polymers have been widely used in medical devices and implants due to their readily controllable chemical, electrical and thermal properties [11] and due to their biocompatibility, unlike alternatives such as metals and ceramics typically used in sensors. These polymers can also be made as soft materials with attractive physical properties due to their controllable Young’s moduli and fabricated readily into a variety of sizes and shapes on demand [12]. Different classes of polymers have been coated onto electrode surfaces to minimise biofouling, through reducing the interactions between the electrode surface and proteins/cells [13]. For example, the hydrophilic polymer polyethylene glycol (PEG) has been shown to form a strong hydration layer which acts as a physical barrier to protein adsorption [14]. Zwitterionic polymers have also been shown to form a strong hydration layers whilst sometimes having the advantages of being biodegradable and non-immunogenic [15]. Conducting polymers, when combined with such polymers, have been used to reduce biofouling; for example, both polypyrrole (PPy) and polyaniline (PANI), when combined with PEG, have been shown to significantly enhance fouling resistance against BSA adsorption [16]. Another study reported an antifouling electrochemical dopamine sensor using the conductive polymer, poly(3,4-ethylenedioxythiophene) (PEDOT) combined with a water insoluble ionic liquid, 1-ethyl-3-methylimidazolium bis(trifluoromethylsulfonyl)imide on glassy carbon electrodes [17]. This enabled the detection of dopamine with a LoD of 33 nM in the presence of human serum. Hydrophobic/fluorous polymers might also hold significant potential as antifouling coatings; for example Xue et al. showed that fluorinated methacrylate-based polymers could be fabricated onto poly(ethylene terephthalate) (PET) fabrics via surface-initiated atom transfer radical polymerisation. This allowed the tuning of wettability based on polymerisation time (polymer length), and were effective in improving surface antifouling properties [18]. Other polymeric coatings such as Nafion [19], polyvinyl chloride (PVC) [20], poly(1,3,5-tris(3-indolcarbonyl)benzene) (PTICBL) [21] and copolymers of a poly(vinylidene fluoride) (PVDF) and poly(oxyethylene methacrylate) (POEM) [22] can act as selective membranes, allowing only the target analytes (or a certain set of molecules with given characteristics) to permeate onto the electrode surface, blocking the transport of non-specific macromolecules, and thus minimising or delaying the onset of biofouling.

Recently, Montiel et al. reported the delayed activation and the enhancement of the anti-biofouling characteristics of bare and enzyme (glucose oxidase)-embedded carbon paste electrodes through coating the surfaces with biocompatible, methacrylate-based pH-responsive “transient” polymers [23]. The capability of these polymeric coatings was characterised using blood and saliva samples in terms of the delayed activation and anti-biofouling enhancement of glucose sensing. This approach was then translated into sensing in other biologically relevant media such as gastric (pH ~ 1.5) and intestinal (pH ~ 6.5) fluids [24].

Poly(meth)acrylate-based copolymer formulations (Eudragit^®^, Evonik Nutrition & Care GMBH Darmstadt-Germany are commonly used for (i) immediate, (ii) delayed and (iii) time-controlled (sustained) drug-release applications, controlled by the formulation, chemical structure and size of the copolymer [25]. Delayed release systems are typically formulated for drugs targeted towards specific components of the gastrointestinal (GI) system, e.g., acid triggered release for drugs that need to be released in the stomach and acid resistant coatings for drugs that would degrade if not protected [26]. Polymers have also been designed to allow delivery to more remote locations in the GI system for targeted clinical purposes; an example is Eudragit^®^ S100 which, as it dissolves at pH > 7.0, has been used to target drug delivery in the ileum [25]. Considering the hydrophobic nature of Eudragit^®^ S100, it can be thought to increase the non-specific protein adsorption on surface and correspondingly resulting in enhanced biofouling [27,28]. However, the dissolution mechanism of the polymer includes the diffusion of water/hydroxide ions into its matrix and chain disentanglement which likely separates the adsorbed proteins and removes them from the surface.

**Figure 1:**
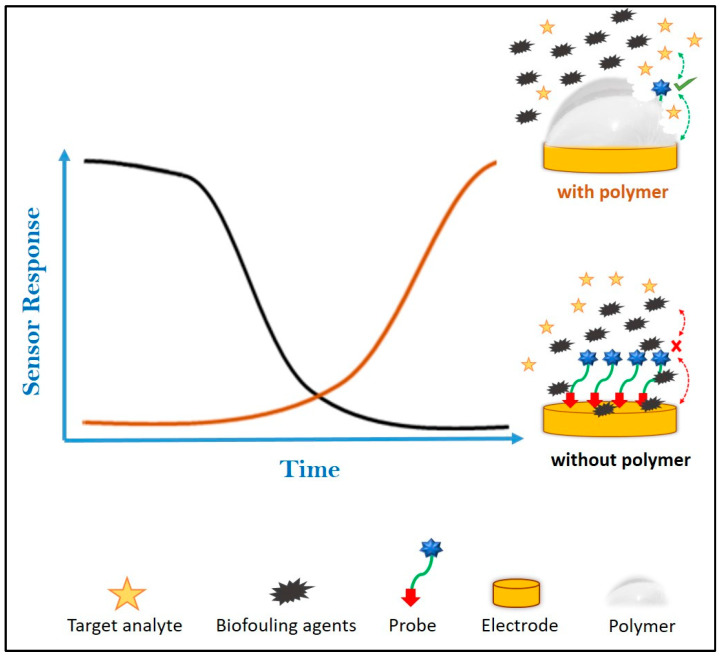
Principle of sensor activation and biofouling protection by the dissolvable polymeric coating that is coated onto the probe-modified electrode.

Here, we investigated approaches to delay and control the activation and the anti-biofouling characteristics of sensors based on functionalised gold screen printed electrodes (Au-SPEs) that had been coated with pH-dissolvable polymers (Figure 1). The sensor systems chosen were based on electrochemical sensors for pH [29] and the activity of the protease trypsin [30,31] with Au electrodes functionalised using self-assembled monolayers (SAM). Here, we compare the performance of the antifouling coatings on bare and SAM-based probe-functionalised electrodes, and discuss the applicability of this work in the area of in vivo sensing. Although the applicability of a similar polymer (Eudragit^®^ L100) has been previously confirmed for enzymatic biosensing [23], this is the first time study where transient polymeric coatings have been shown to enhance anti-biofouling characteristics of SAM-modified electrodes.

## 2. Materials and Methods

### 2.1. Instrumentation

All electrochemical measurements were carried out using a conventional three-electrode electrochemical cell which was driven by a personal computer-controlled AutoLab PGstat-30 potentiostat running the NOVA 2.1 software (Metrohm Autolab B.V., Utrecht, The Netherlands). Commercially available (Metrohm DropSens, C220AT) screen printed electrodes (SPEs, working electrode: Au, auxiliary electrode: Au, reference electrode: Ag) were used for measurements. Those with a working electrode (WE) of gold are denoted Au-SPE. The working electrode potential, *E*, was applied with respect to, and is reported relative to, the screen-printed Ag pseudo reference electrode. For implantable sensing relevance, a Lauda Eco Silver thermostatic bath (VWR International Ltd., Lutterworth, UK) with an external pumping system and a water-jacketed glass cell was used to control the temperature of all the experiments conducted at body temperature (37 °C). pH values were measured using a Fisherbrand Hydrus 400 pH meter (Thermo Fisher Scientific, Oxford, UK). For surface morphology characterization of bare and polymer-coated electrodes, Scanning Electron Microscopy was performed using a HITACHI SU1000 FlexSEM 1000II instrument. Before SEM imaging, a ~50 nm Au layer was deposited on polymer coatings to avoid charging issues.

### 2.2. Reagents and Materials

Eudragit^®^ S100 (molecular weight, MW ~125 kDa [32]) was supplied, in powder form, by Evonik Nutrition & Care GMBH. Dulbecco’s Modified Eagle Medium (DMEM), fetal bovine serum (FBS), ethanol, isopropanol, potassium ferri/ferrocyanide (FFC), disodium phosphate and 10× PBS were purchased from Sigma-Aldrich (UK) and used as received. All reagents were of analytical grade and all solutions were prepared using protease-free deionised water.

### 2.3. Synthetic Methods

The experimental procedures for the synthesis and characterisation of the methylene blue (MB)-labelled pH and protease (trypsin) probes were detailed in previous studies [29,30].

### 2.4. Cleaning and Preparation of Electrodes

Au-SPEs were initially subjected to electrochemical cleaning by carrying out cyclic voltammetry (CV), performing potential cycles between 0 and +1.6 V in 0.1 M H_2_SO_4_ at a scan rate of 100 mV·s^−1^ until the characteristic voltammogram of clean gold was obtained. The probe molecules (probe-1: pH or probe-2: trypsin) were immobilised on the cleaned working electrode surface by drop-casting 10 μL of a 40 μM ethanolic probe solution overnight at 4 °C. Remaining non-immobilised molecules were removed by performing two sequential washings in ethanol and PBS. The Au-SPE working electrode surfaces (unmodified or probe-modified) were coated with the pH responsive polymer Eudragit^®^ S100 by dissolving the polymer in isopropanol at three different concentrations (8, 16 or 32% (*w*/*v*)). 10 μL of this solution was then drop-cast onto a probe-modified or an unmodified (bare) electrode surface, either as a single layer or by repeated drop-casting to give double or triple consecutive layers. After drop-casting, the isopropanol solvent was left to evaporate to dryness at room temperature for >2 h before use. If stored, these modified electrodes were stored at 4 °C until use.

### 2.5. Electrochemical Characterisation of Polymer Dissolution

Electrochemical characterisation of polymer-coated electrodes was performed in order to assess the delay in exposure of the redox tagged probe-modified and unmodified electrode surface and the anti-biofouling capabilities. Polymer-coated electrodes were incubated in PBS (characterisation-optimisation of the probe-modified electrodes) or 5 mM potassium ferri/ferrocyanide (FFC) in PBS (an external redox agent to allow electrochemical monitoring of the degree of exposure of the underlying bare electrode) or 10% FBS in DMEM solutions for biofouling characterisation. These electrodes were subjected to cyclic voltammetry (CV, at a scan rate of 100 mV s^−1^) and square wave voltammetry (SWV, applying *E* at a frequency of 60 Hz, with an amplitude of 25 mV and a step potential of 5 mV), performing successive scans with time to monitor the dissolution of the coated polymer layers. For both CV and SWV measurements, the redox tag methylene blue potential window (−0.05 to −0.4 V vs. Ag) was selected for the probe-modified electrodes and the FFC potential window (0 to 0.5 V vs. Ag) for the unmodified electrodes. In all experiments, the redox signal (peak current height, *i_p_*, at a fixed redox potential corresponding to either methylene blue or FFC) was monitored with time, *t*, from its initial value (*t* = *t*_0_) until this reached a stable value and did not change any further with time (*t = t_∞_*, corresponding to the complete dissolution of the polymer coating). The resulting signal at time *t* was expressed as

$$Signal\;\left(t\right)=\frac{i_{p}\left(t\right)-i_{p}\left(t_{0}\right)}{i_{p}\left(t_{\infty }\right)-i_{p}\left(t_{0}\right)}\times 100\%$$

with the initial redox signal defined as 0% and the final signal as 100% in all cases, so that the signal showed the effectiveness of the barrier polymer layer at all times.

## 3. Results and Discussion

We previously reported two electrochemical sensing platforms based on the formation of SAMs of redox-tagged (methylene blue) probes on gold or platinum electrodes for the detection of pH changes in vivo [29] and protease (trypsin) activity in vitro [30,31]. Herein, we develop the use of a protection strategy based on a pH-activated dissolvable polymer coating drop-cast on top of these probe-modified electrodes, allowing the controlled, delayed exposure of the sensing phase leading to improved anti-biofouling performance when compared to non-coated sensors.

### 3.1. Characterisation of Polymer Dissolution

**Figure 2:**
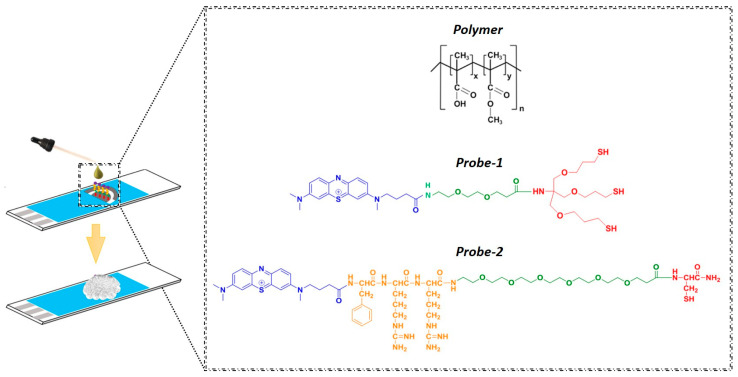
The preparation of the sensing electrode. Chemical structure of Eudragit^®^ (top): The ratio of methacrylic acid and methyl methacrylate units was approximately 1:2 for Eudragit^®^ S100 (n is the degree of polymerisation). The chemical structures of the probes used for pH (Probe-1) and trypsin (Probe-2) sensing: Probe-1 contains methylene blue (blue) as the redox tag, PEG-2 as a spacer and a tripodal anchor (red), whereas Probe-2 contains methylene blue (blue) as the redox tag, attached to the trypsin-cleavable peptide sequence Phenylalanine-Arginine-Arginine (orange) and PEG-6 (green) as the spacer and Cysteine (red) as the anchor.

Eudragit^®^ S100 (molecular weight, MW ~125 kDa) is a solid, anionic copolymer consisting of methacrylic acid and methyl methacrylate in a ratio of 1:2 (Figure 2) [33]. The mechanism behind the pH-dependent dissolution of Eudragit^®^ S100 polymers was previously explained by Nguyen et al. as occurring in five steps, including the diffusion of water/hydroxide ions into the polymer matrix, ester hydrolysis, gel layer formation, polymer chain disentanglement, and further chain ionisation and dissolution [34]. The main reason behind the pH-responsive nature of Eudragit^®^ S100 is the presence of the weakly acidic carboxyl groups which are initially in the “protonated” form at acidic pH’s and then start to undergo dissociation as the pH increases (the pKa value of Eudragit type S is ~6), with the negatively charged carboxylate groups then promoting disentanglement and polymer diffusion into bulk solution [35]. In addition, at higher pH’s, there is increased hydrolysis of the ester groups that produces additional carboxylate groups which further promote the rate of disentanglement.

**Figure 3:**
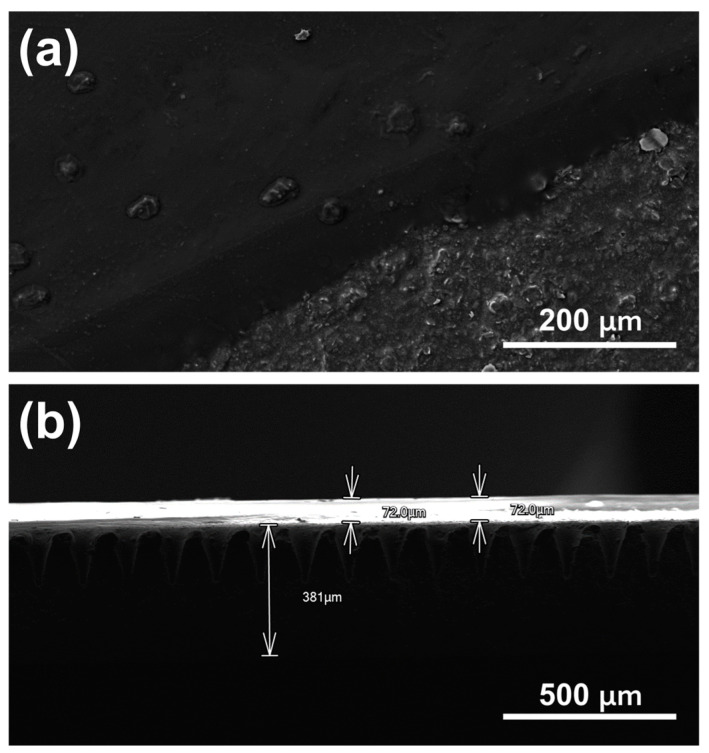
(**a**) Top-view and (**b**) cross-sectional SEM images of the polymeric film coating.

**Figure 4:**
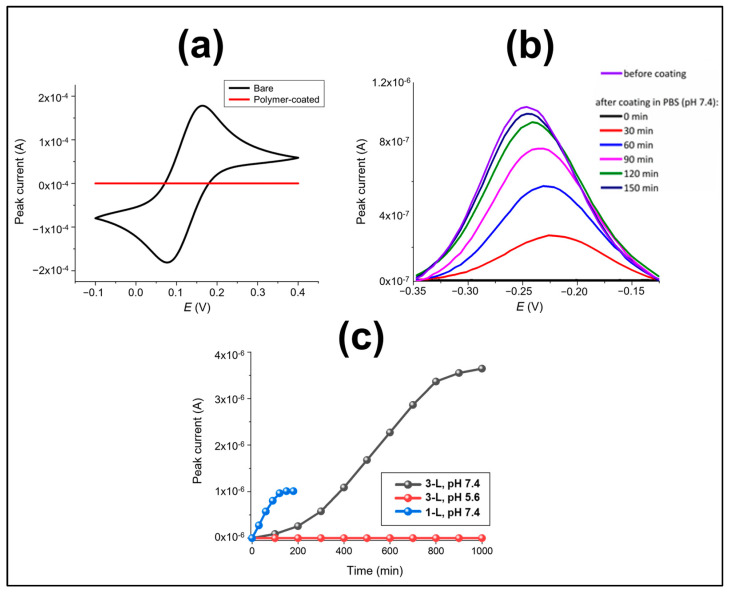
Analysis and characterisation of polymer film integrity and dissolution: (**a**) CVs of Au-SPE recorded in 5 mM potassium ferri/ferrocyanide in PBS, before (black) and after (red) coating with a single layer of 16% Eudragit^®^ S100. (**b**) SWV curves recorded for an Au-SPE modified with Probe-1 and then coated with a single layer of the 16% Eudragit^®^ followed by its immersion in PBS. (**c**) SWV peak current vs. time registered for an Au-SPE modified with Probe-1 and then coated with a single layer (blue, Figure 4b) or three layers of 16% Eudragit^®^ S100 when incubated in PBS at pH 5.6 (red) and at pH 7.4 (black) for 1000 min.

SEM imaging was first performed for morphological characterisation of the bare and polymer-coated regions as shown in Figure 3. It was observed that the surface roughness did not significantly increase on coated areas due to polymer film uniformity, where its thickness was found to be ~72 μm. In order to confirm that complete polymeric blocking of bare electrode activity was achieved, a polymer layer integrity test was performed by recording cyclic voltammograms between −0.1 and +0.4 V at a scan rate of 100 mV/s in 5 mM FFC in PBS (Figure 4a). Comparison of polymer-coated with the uncoated bare electrodes showed the complete removal of the FFC redox peaks confirming a polymer coating of high integrity with effective blocking of the electrode surface.

Figure 4b then shows typical SWV curves registered for an Au-SPE which had been modified with the pH-sensing probe (Probe-1) ([29], drop-casting overnight) compared to that obtained after coating with a single layer of 16% Eudragit^®^ S100. It is clear that the SWV peak current (purple curve) of ~1.1 µA before the polymer layer was drop-cast, decreased to zero after the polymer was coated on top of the probe-1-modified electrode surface, again confirming that the polymer layer completely covered the SAM-modified surface and completely blocked the redox reaction of the underlying surface-bound redox tags. This was attributed to the effects of this resistive polymer layer in blocking electron transfer by preventing solvation, and ion/proton transport to the methylene blue (MB) from the solution. As expected, at pH 7.4, the characteristic redox peak for MB was observed and this signal increased with time due to progressive dissolution of the polymer layer. The signal essentially returned to the initial, uncoated value within 150 min, indicating ~95–100% dissolution. It was also observed that the dissolution rate was faster initially and then levelled-off over time. It is interesting that there was a progressive shift of ~−30 mV in the SWV peak potentials between the partially (after 30 min dissolution) and fully uncoated probe-modified electrode, albeit with a similar peak width consistent with a clear difference in the average redox environment, most likely due to attributed to the local ion activity and/or solvation environment for the SAM-based probes.

In order to confirm the pH specificity and indicate the potential for time control of the dissolution of Eudragit^®^ S100 coatings, two Au-SPEs were modified with Probe-1 and each coated with three consecutive layers of 16% Eudragit^®^ S100. They were then subjected to SWV measurements between 0 and −0.4 V (the methylene blue redox potential window) in PBS solutions at (i) pH 5.6 and (ii) pH 7.4 for 1000 min. As shown in Figure 4c, there was no redox signal observed for the electrode immersed at pH 5.6, whereas the peak current for the electrode immersed in pH 7.4 showed progressive film dissolution and underlying electrode exposure over the 1000 min period. This is consistent with previous findings [36] where it was reported that combinations of the polymers L100 and S100 with greater than 50% of S100 did not release any of the model drug, mesalazine at pH 6.5 PBS. In addition Eudragit^®^ S100 has already been shown to dissolve above pH 7.0 and is employed for drug release in the colon for the treatment of diseases such as ulcerative colitis, Crohn’s disease, and irritable bowel syndrome [37]. This result further confirms that its activation/dissolution is highly specific to pH. In addition, changes in peak currents when only a single layer of coating was applied (data shown in Figure 4b) was plotted against time and compared with the dissolution curve of the triple layer, showed that the delay time can be also controlled (Figure 4c). Thus, optimisation of the polymer coating on electrode surfaces, with changes in the number of coated layers and their concentration will allow subtle control of electrode exposure time and rates.

### 3.2. Optimisation of Polymer Coating Formation (Thickness and Concentration)

It has been shown that dissolution times of the Eudragit polymer layers vary depending on structural factors such as the thickness of the coating layers and the morphology of the cast layer, which can itself be varied by changing the concentration of the drop-cast polymer solution [23]. However, this has never been performed for any SAM-modified electrode surfaces. Therefore, it was important to understand how thickness and concentration affected the polymer dissolution time and performance in this case, in order to assess the potential for control of these parameters for in vivo SAM-based sensor protection applications.

**Figure 5:**
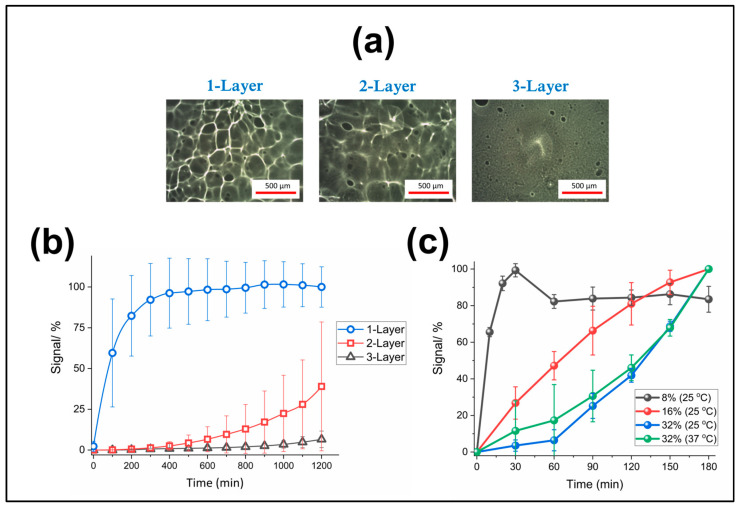
Sensor optimisation: (**a**) Optical microscope images of the Au-SPEs drop-cast with 1, 2, or 3 consecutive layers of 16% (*w*/*v*) of Eudragit^®^ S100 in isopropanol. (**b**) The relationship between % signal change and polymer dissolution time and layer thickness, as measured by the redox signal for Probe-1 attached to the Au-SPEs and coated with different numbers of polymer layers (1, 2 or 3-L) upon immersion in PBS (pH 7.4) over 1200 min and interrogated by SWV. (**c**) The relationship between the % normalised change in the redox signal registered for Probe-1 modified Au-SPEs drop-cast with a single layer of Eudragit^®^ (from solutions with varying concentrations of 8%, 16% or 32% *w*/*v*) during immersion in PBS at the specified temperature over 3 h and interrogation with SWV. In all cases the averages and error bars are for three replicates.

The Influence of film thickness on polymer dissolution time was therefore assessed for probe-1-modified Au-SPEs, which were coated with a controlled number of layers of the 16% (*w*/*v*) solution. Figure 5a shows resulting optical microscopy images of a typical region of the coated area for each electrode (one, two or three layers). It is clearly seen that increasing the layer thickness resulted in a more uniform polymer coating, with a reduced number of surface defects such as holes and bumps (these images were selected from a number of randomly chosen areas as being representative of and comparable to other images recorded across the whole electrode surface). As such, there was a significant variation between identically prepared electrodes, when coated with one or two layers due to these variable film defects, as represented by the optical images shown in Figure 5a. This variability was reduced for the electrode modified with three layers of the coating, due to the filling of the defects in the underlying films. Figure 5b shows the corresponding percentage signal change vs. time registered by SWV for these different Au-SPEs Probe-1 modified electrode with one, two or three layers of 16% Eudragit polymer coating. It was observed that polymer dissolution (electrode activation) could be delayed by more than 20 h when using three layers of the polymeric coating, while one layer of the coating allowed exposure of the sensor surface within 2–8 h.

The influence of the polymer concentration of the mix used for the drop-cast coating was analysed by employing different Au-SPEs each identically modified with Probe-1 and then coated with single layers of polymer mix which contained different weight to volume percentages (8%, 16% or 32%) of the S100 polymer (because the drop-cast volumes (10 μL) was kept constant, the deposited amount/thickness of polymer film increased). Figure 5c shows the dependency of the resulting polymer dissolution time on the polymer concentration. For the concentrations 8% and 16%, the exposure rate was observed to be linear (within experimental error). The polymer layers coated from solution with a concentration of 8% completely dissolved in only 30 min, while it was observed that although both 16% and 32% coatings were both fully exposed after approximately 3 h. However, the initial activation rate was markedly slower for the 32% polymer coating prepared presumably due to the thicker layer of polymer deposited.

When the 32% coating was tested at 37 °C (in order to understand whether temperature has an effect on the dissolution rate), an increase in temperature resulted in an enhanced dissolution rate over the first 60 min, but this then levelled off and was found not to be significant over longer frame time points. In summary, these results show the potential to control polymer dissolution times, and the ability to delay the extent of sensor activation by fine-tuning the polymer preparation/fabrication parameters.

### 3.3. Biofouling Protection

**Figure 6:**
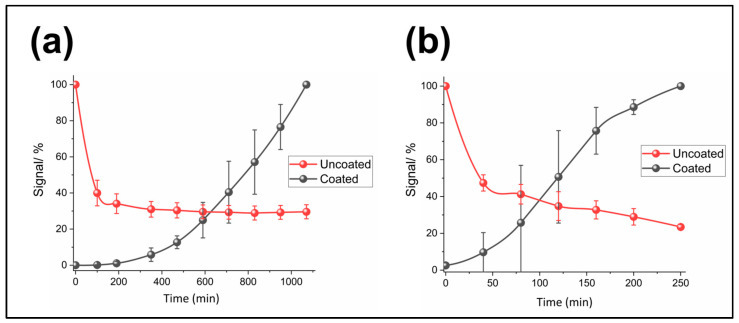
Anti-biofouling performance of the sensor: (**a**) % Signal changes measured for the sensor coated with three consecutive layers of 16% Eudragit^®^ and the uncoated sensor (Au-SPEs modified with Probe-1) in DMEM with 10% FBS (pH ~7.2). (**b**) % Signal changes measured for the sensor coated with a single layer of 16% Eudragit^®^ and the uncoated sensor (Au-SPEs modified with Probe-2) in DMEM with 10% FBS. The averages and error bars are for three replicates.

After demonstrating the ability to control the delay in sensor activation, the anti-biofouling protection characteristics of these polymeric coatings were investigated. To achieve this, polymer-coated (or uncoated as a control) pH and trypsin sensors were prepared on Au-SPEs and tested at 37 °C in Dulbecco’s Modified Eagle Medium (DMEM) including 10% fetal bovine serum (FBS) which mainly consists of proteins such as BSA and is commonly used for in vitro biofouling characterisation [38]. The anti-biofouling protective properties (Figure 6) were evaluated by comparing electrochemical signals from polymer-coated and uncoated samples over time and determining the pH or trypsin sensing performance of the polymer-coated sensors after sufficient time to complete polymer dissolution.

The development and in vitro and in vivo characterisation of the MB-based pH sensors can be found elsewhere [29]. Briefly, the sensor was developed by depositing a SAM-functionalised film of probe molecules (Probe-1) onto microfabricated three-electrode sensors in order to monitor tumour microenvironment pH in real-time based on the established pH-specific variation of the redox behaviour of MB [39]. In vitro test results exhibited a linear dependency between the peak potential of the MB reduction and the pH, giving −26 mV/pH; the expected Nernstian behaviour for a 2e^−^/1H^+^ redox process. Some of the sensors also included Nafion as a top layer on these probe-modified electrodes given that it is a widely used cation-exchange polymeric membrane. The use of Nafion resulted in better pH sensitivity, showing more than a two-fold change in the calibration line (−68.0 mV/pH). This was attributed to Nernstian behaviour for a 2e^−^/2H^+^ redox process due to a shift in the pKa of reduced MB arising from the negatively charged nature of the Nafion membrane. Although the sensor system exhibited promising results when tested in vivo through implantation into ovine pulmonary adenocarcinoma (OPA) tissue [29,40], it also established that better anti-biofouling protection approaches are needed for longer-term measurements and why transient polymeric coatings were investigated here.

Figure 6a shows the relative changes in the SWV peak current of the polymer-coated and uncoated Probe-1 pH sensor system over 1000 min. The signal registered for the uncoated sensors decreased rapidly by 65–70% within this time frame, consistent with non-specific binding reducing the electrochemical signal, whereas the polymer coated sensors showed a signal which continuously and reproducibly increased with time. Because the polymer-coated sensors are not likely to be fully exposed after 1000 min (less than 10% of the signal was recorded with three polymer layers after 1200 min) as shown in Figure 5b, the relative signal changes were normalised with respect to the final measured signals (instead of the expected 100% signal). This means that some polymer will still be on top of the probe molecules without full exposure, but with some open channels allowing redox interactions. While the dissolution was still occurring, the fact there was not any indication of signal decrease due to non-specific protein binding onto these coated surfaces on this timescale clearly suggests enhanced anti-biofouling, provided by these transient polymeric coatings.

Similar to the anti-biofouling characterisations performed for the pH sensors, the trypsin sensors were also prepared (modifying Au-SPEs with Probe-2 and either coated with a single layer of 16% Eudragit^®^ on top or uncoated (control)) and were subjected to SWV measurements in DMEM including 10% FBS. Figure 6b shows the relative changes in the SWV peak current of the polymer-coated and uncoated trypsin sensors over 250 min (in this case, ~3–4 h was enough to obtain an exposed surface (Figure 5b)). The signal registered for the uncoated sensors decreased by 70–75% after 250 min whereas a continuous increase was observed for the coated surfaces. A signal decrease of ~60% was obtained during the first 100 min for the uncoated sensors and was consistent and similar for both pH and trypsin sensor experiments, suggesting that most of the non-specific protein adsorption takes place during this period. As expected, the signal registered for the polymer-coated trypsin sensors increased over 250 min although there are some variations between the replicates. These variations were similar in magnitude and can again be related to the non-uniformity of the single polymer layer (Figure 5a), which might have caused inhomogeneous dissolution of the coating layer surface. It was interesting that the curve of signal increase observed for the coated surfaces was S-shaped, rather than the linear response observed in Figure 5b,c. This might suggest the formation of biofouling on top of the polymer, inhibiting its initial, or even longer-term dissolution. This is also consistent with the thought that the dissolution mechanism is based on the opening of channels which permit solvent/ion transmission to the active parts of electrode (with some remaining polymer), rather than complete dissolution from the surface. Although further studies are necessary for understanding the whole mechanism, the results show that the dissolvable polymeric coatings improved the anti-biofouling characteristics of these sensors. We believe that the ideas suggested/explored in this study can be applied to any SAM-based sensor/biosensor recognition probes. Some other recent approaches used to protect electrochemical sensors against biofouling in comparison to this study have been summarized in Table 1. As a possible limitation of our study, some further analyses are required to assess the biocompatibility of the polymer for in vivo applications.

### 3.4. Analysis of Post-Dissolution Sensor Performance

**Figure 7:**
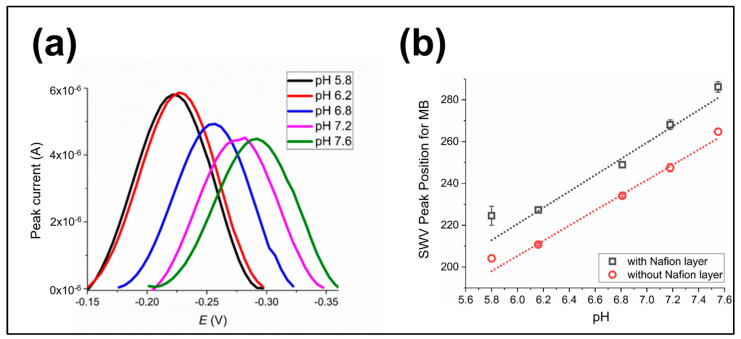
Characterisation of the post-dissolution pH sensor: (**a**) SWVs with a Nafion-coated pH sensor immersed in phosphate buffers of varying pHs: 5.8 (black), 6.2 (red), 6.8 (blue), 7.2 (magenta) and 7.6 (green). (**b**) Calibration line of the MB redox pH sensor potentials (average of reduction and oxidation) registered for the Nafion-coated and uncoated sensors versus pHs measured with a glass electrode. All data points represent the average and standard deviation for 3 replicates and the straight lines correspond to the best linear fit.

It was important to show that the protected and activated sensors had similar performance before and after polymer dissolution. Therefore, an experiment was performed to check the pH sensitivity of the exposed sensors, using the same method as previously reported [29]. As shown in Figure 7a, Nafion-coated and uncoated pH sensors (following the complete dissolution of the top Eudragit^®^ S100 layer) were placed in phosphate buffers with varying pHs (5.8, 6.2, 6.8, 7.2 and 7.6) and interrogated by SWV. Afterwards, the peak potential of the MB redox signal was plotted against pH to create a calibration line for their pH response (Figure 7b). This resulted in pH sensitivities of 36 ± 2 mV/pH unit for Nafion-uncoated and 39 ± 4 mV/pH unit for Nafion-coated sensors. Considering the same Nafion coating protocol (drop-casting 3 μL water-diluted Nafion solution at 1:3 ratio) used as in previous work [29], it was interesting that here Nafion did not show any significant difference in potential-pH sensitivity, rather a difference in potential offset, and the slope observed was midway between 2e^–^/2H^+^ and 2e^–^/1H^+^ behaviour, which could be an indication of the possible interaction between Nafion and the Eudragit^®^ S100 polymers altering the redox behaviour of the MB.

## 4. Conclusions

Herein, the applicability of adding dissolvable pH-activated commercial polymeric coatings onto redox-tagged SAM-modified electrode surfaces developed for measuring pH and trypsin activity were evaluated in terms of their anti-biofouling characteristics. An enhancement of the anti-biofouling properties of both of the sensing platforms, particularly after short incubation times (100 min) were observed when tested in biologically relevant medium. This delay in sensor exposure and activation was found to be vital for avoiding the effects of rapid, non-specific, protein adsorption. By optimising the thickness and concentration of the coated polymeric layer, delay times could be increased to up to 20 h with a potential for further increases possible by thicker polymer layers or different polymers. The analytical performance of the polymer-coated SAM-based pH sensor was characterised before and after complete polymer dissolution. Although the sensitivity of Nafion-coated pH sensors apparently slightly decreased, the sensor platform was still redox-active and sensitive to pH changes. Together, these promising findings offer potential for translation for in vivo sensing, and future work will focus on ways to further increase and control activation times and to integrate these coatings with in-house built integrated devices.

## Figures and Tables

**Table 1:** Recent approaches used for sensor protection against biofouling.

Material	Electrode Type	Analyte	Significant Remarks	Ref.
Poly(trifluoroethyl methacrylate-random-sulfobetaine methacrylate) (PTFEMA-r-SBMA)	Solid-state ion-selective electrodes	NH_4_^+^	Highly sensitive and long-term stable sensing performance was shown in real wastewater for 55 days.	[41]
Liquid-like polydimethylsiloxane (PDMS)	Integrated Au electrodes	Reactive oxygen species	Stable sensing performance was observed after 3 days of incubation with bacteria and sensitive ROS detection in bacteria-rich media over 24 h was achieved.	[42]
Ag NPs/ hydrophilic polydopamine	Glassy carbon electrodes with ion selective polymer membrane	Na^+^, Ca^2+^, Mg^2+^, Li^+^, Ag^+^	Due to the anti-bacterial properties of Ag NPs, the sensor showed good sensing ability even after contact with bacterial suspension for 7 days.	[43]
Methacrylic acid and methyl methacrylate copolymer (Eudragit^®^ L100)	Bare carbon or GOx-PB-graphite SPEs	Glucose	Controlled sequential sensor activation was found to delay biofouling for enzymatic glucose sensing in blood and undiluted saliva samples over a 2 h period.	[23]
Methacrylic acid and methyl methacrylate copolymer (Eudragit^®^ S100)	Au SPEs	pH and protease	Up to 20 h delay against biofouling effects was achieved for electrodes with SAM-probes.	This study

## Data Availability

The data presented in this study are available on request from the corresponding authors.
